# A machine learning model for predicting bone and/or lung metastasis in differentiated thyroid carcinoma: enhancing precision in risk stratification

**DOI:** 10.3389/fendo.2025.1528392

**Published:** 2025-09-08

**Authors:** Libin Huang, Limei He, Ru Chen, Shengyin Liao

**Affiliations:** ^1^ Department of Medical Oncology, The First Hospital of Putian, Teaching Hospital, Fujian Medical University, Putian, Fujian, China; ^2^ The School of Clinical Medicine, Fujian Medical University, Fuzhou, Fujian, China; ^3^ Department of General Surgery, The First Affiliated Hospital of Nanchang University, Nanchang, China

**Keywords:** thyroid cancer, bone metastasis, lung metastasis, SEER, machine learning

## Abstract

**Background:**

Differentiated thyroid cancer (DTC) incidence is rapidly rising worldwide. While most cases have a favorable prognosis, a subset of patients develop aggressive disease with distant metastases, particularly to the bone and lung, which significantly worsens outcomes. Current prediction models are limited in accuracy, often relying on basic clinical factors. This study aims to develop a machine learning model to improve prediction of bone and lung metastasis in DTC, enhancing risk stratification and early intervention.

**Methods:**

Using the SEER database, we developed several machine learning models—including XGBoost, Random Forest, Gradient Boosting Machine, Logistic Regression, Naive Bayes, and Classification and Regression Trees (CART)—to predict bone and lung metastasis risk in DTC patients. LASSO regression was applied to select key predictive variables, and SMOTE was used to address data imbalance. The model’s generalizability was evaluated using an external validation cohort from China.

**Results:**

The XGBoost model demonstrated the highest performance, achieving an AUC of 0.988. Key predictive variables identified and included in the model were tumor size, radiation therapy, surgical interventions, histologic types, T and N stages, laterality, race, and household income. SHAP analysis confirmed the importance of these variables, with tumor size, radiation, and surgery emerging as primary predictors. In the external validation cohort, the model achieved an AUC of 0.866, indicating reliable predictive capability across clinical settings.

**Conclusion:**

This model accurately predicts bone and lung metastasis risk in DTC, offering valuable clinical utility for risk stratification and supporting early intervention strategies to improve outcomes in high-risk patients.

## 1 Induction

Thyroid cancer (TC) is one of the most rapidly increasing malignancies globally, with a notable rise in incidence over the past few decades (1, 2).Differentiated thyroid cancer (DTC) is the most common type of malignant thyroid tumor, originating from the follicular epithelial cells of the thyroid. It accounts for a significant portion of endocrine cancers, and although the prognosis for most patients is generally favorable, a subset presents with aggressive disease characterized by distant metastasis (3). Specifically, bone and lung metastases are among the most common sites, contributing substantially to morbidity and mortality among DTC patients. The presence of distant metastases at diagnosis or during follow-up dramatically worsens the prognosis and reduces overall survival, underscoring the importance of early and accurate identification of patients at risk.

Despite advances in diagnostic and therapeutic approaches, current strategies for predicting metastasis in DTC remain suboptimal. Most existing prediction models rely on a combination of clinical factors, such as tumor size, age, and histologic features, but these approaches often fail to comprehensively capture the complex, multifactorial nature of metastasis development (4, 5). Additionally, traditional risk stratification relies heavily on subjective clinician judgment and limited clinical data, leading to challenges in generalizability and accuracy. Consequently, there is a clear unmet need for robust, reproducible models that incorporate diverse clinical features to improve the identification of high-risk individuals who may benefit from more intensive surveillance or early intervention.

Recent advancements in machine learning have offered promising avenues for improving prediction models in oncology. Machine learning techniques allow for the simultaneous evaluation of numerous variables and can uncover non-linear relationships within high-dimensional datasets, providing a more nuanced assessment than conventional statistical models (6–8). However, the application of machine learning to predict metastatic risk in DTC is still in its nascent stages, and few studies have leveraged the power of ensemble learning and external validation to enhance model reliability. Additionally, the inherent imbalance in datasets, where metastatic cases are significantly fewer compared to non-metastatic cases, poses a challenge to many predictive models, often resulting in suboptimal sensitivity and false-negative results.

In this study, we aimed to address these gaps by developing a comprehensive machine learning model to predict bone and/or lung metastasis in patients diagnosed with thyroid cancer. Utilizing the SEER database, we constructed a retrospective cohort to identify clinical predictors associated with metastatic risk. Our study also included an independent external validation cohort from a clinical population in China to evaluate the generalizability of the model across different settings. The objective of our research is not only to improve the prediction accuracy of metastatic risk in DTC but also to provide an accessible tool that integrates seamlessly into clinical workflows. By leveraging machine learning techniques, our study aims to fill the existing gaps in metastasis prediction, improve patient stratification, and ultimately contribute to enhanced clinical decision-making in the management of thyroid cancer.

## 2 Method

### 2.1 Data sources and study population

**Figure 1:**
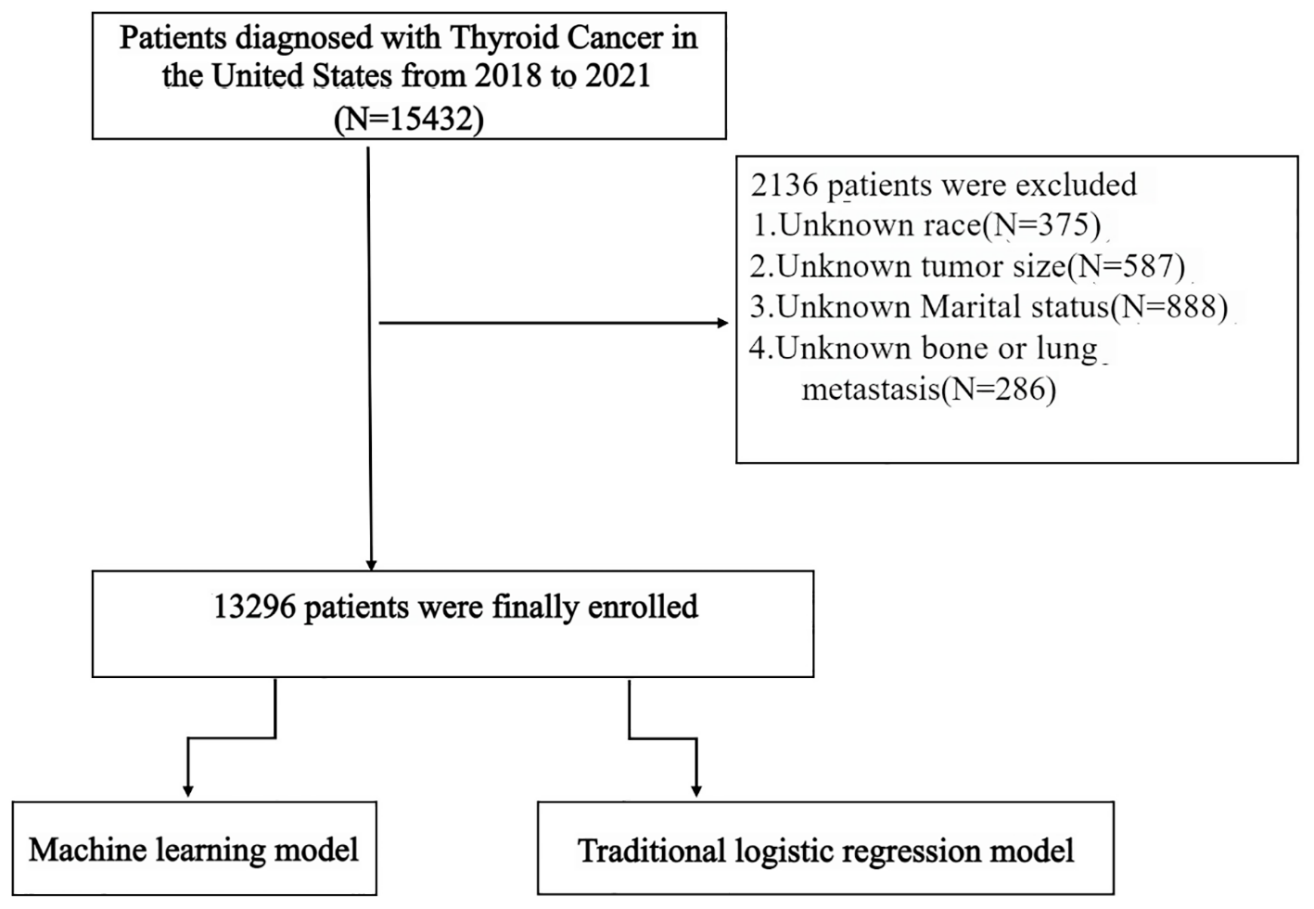
Study cohort selection flowchart.

This retrospective study utilized data from the SEER database to identify patients diagnosed with DTC between 2018 and 2021. The SEER database offers extensive, nationwide clinical and demographic information, serving as a valuable resource for population-based epidemiological studies. The initial cohort comprised 15,432 DTC patients. However, 2,136 patients were excluded due to missing critical information, specifically including unknown race (N = 375), unknown marital status (N = 888), and unknown data on bone or lung metastasis (N = 286), as illustrated in **Figure 1**.

An external validation cohort of 255 DTC patients diagnosed at the First Affiliated Hospital of Nanchang University and the First Hospital of Putian during the same period (2018–2021) was incorporated to assess the model’s generalizability and performance. All patients in the external validation cohort had confirmed DTC diagnoses and complete clinical data for the variables included in the model.

### 2.2 Risk factor screening and model construction

We employed the Least Absolute Shrinkage and Selection Operator (LASSO) regression technique to identify critical clinical predictors for bone and lung metastases. LASSO applies an L1 penalty to the regression coefficients, effectively zeroing some while highlighting others that are most influential in predicting the outcome. This method is particularly advantageous for high-dimensional datasets as it helps reduce multicollinearity and enhances the clarity of the model. Our analysis incorporated an array of clinical variables, including patient age, race, year of diagnosis, sex, laterality, histologic types, T and N stages, radiation treatment, surgical interventions, chemotherapy status, tumor size, marital status, and household income. We optimized the regularization parameter λ using 10-fold cross-validation to minimize prediction error and prevent overfitting. The most significant features identified for subsequent model development included radiation treatment, surgical interventions, tumor size, histologic types, N stage, laterality, T stage, race, and household income.

### 2.3 Model construction and model performance evaluation

To predict bone and/or lung metastasis in differentiated thyroid carcinoma (DTC) patients, we constructed and compared six supervised machine learning algorithms: Logistic Regression (LR), Random Forest (RF), Gradient Boosting Machine (GBM), Extreme Gradient Boosting (XGBoost), Naive Bayes (NB), and Classification and Regression Trees (CART). All models were implemented in Python 3.9 using scikit-learn 1.2.2 and XGBoost 1.7.3 packages.

The SEER dataset was randomly split into a training set (70%) and test set (30%), stratified by metastasis outcome to preserve class distribution. Categorical variables were one-hot encoded, and continuous variables were standardized (z-score normalization).

Patients with missing values in any included variable were excluded from the analysis. Due to the low prevalence of distant metastases, we applied SMOTE (Synthetic Minority Over-sampling Technique) to the training data only to avoid data leakage. This technique generated synthetic minority samples by interpolating between k-nearest neighbors (k=5), ensuring a balanced class distribution for model training.

Each model underwent 5-fold cross-validation on the training set for hyperparameter tuning via grid search. The optimal hyperparameters for each algorithm were:Random Forest: n_estimators=200, max_depth=10, min_samples_split=4;XGBoost: learning_rate=0.1, n_estimators=300, max_depth=6, subsample=0.8, colsample_bytree=0.8;GBM: learning_rate=0.05, n_estimators=250, max_depth=4;Logistic Regression: penalty=‘l2’, solver=‘liblinear’, C = 1.0;CART: max_depth=5, min_samples_split=10;Naive Bayes: default scikit-learn implementation (GaussianNB).Model training was conducted using the full training set with these optimized parameters. Each trained model was evaluated on the internal test set using the following metrics:Accuracy,Sensitivity (Recall),Specificity,F1 Score,Area Under the ROC Curve (AUC),Precision-Recall Curves,Calibration Curves. We further tested model generalizability using an external validation cohort of DTC patients from the First Affiliated Hospital of Nanchang University and the First Hospital of Putian, applying the same preprocessing and model configuration. Evaluation metrics were re-computed to assess performance in a real-world setting outside the SEER registry To enhance interpretability, we applied SHapley Additive exPlanations (SHAP) to the XGBoost and RF models. SHAP values quantify the contribution of each input variable to model predictions, enabling both global importance ranking and individual-level interpretability.

## 3 Results

A total of 13296 DTC patients from the SEER database were included in this study, of whom 263(1.98%) presented with bone and/or lung metastasis, while 13033(98.02%) had no evidence of metastasis. The external validation cohort consisted of 255 patients diagnosed with DTC at the First Affiliated Hospital of Nanchang University and the First Hospital of Putian between 2018 and 2021, 32(12.55%) of whom had bone and/or lung metastasis. Detailed cohort information is presented in **Table 1**. **Table 2** summarizes the baseline characteristics of DTC patients with and without bone and/or lung metastasis. Significant differences were observed between the groups in several key areas.

**Table 1:** Baseline characteristics of thyroid cancer patients from SEER database and external validation cohort.

Categories	Training set (N = 13296)	Validation set (N = 255)	*P-*value
Age (years)			
mean (SD)	50.64 ± 16.32	51.38 ± 16.28	0.22
Sex			0.533
Female	9697 (72.93%)	181 (70.98%)	
Male	3599 (27.07%)	74 (29.02%)	
Marital status			0.552
Divorced	930 (6.99%)	17 (6.67%)	
Separated	101 (0.76%)	4 (1.57%)	
Unmarried	159 (1.2%)	1 (0.39%)	
Widowed	562 (4.23%)	11 (4.31%)	
Married	8353 (62.82%)	156 (61.18%)	
Single	3191 (24%)	66 (25.88%)	
Household income			0.368
<$60000	1748 (13.15%)	27 (10.59%)	
$60000 - $79,999	2780 (20.91%)	47 (18.43%)	
$80000 - $119,999	8110 (61%)	169 (66.27%)	
$120,000+	658 (4.95%)	12 (4.71%)	
Year of diagnosis			0.731
2018	3428 (25.78%)	65 (25.49%)	
2019	3476 (26.14%)	60 (23.53%)	
2020	2979 (22.41%)	63 (24.71%)	
2021	3413 (25.67%)	67 (26.27%)	
Laterality			0.543
Bilateral	155 (1.17%)	2 (0.78%)	
Left	384 (2.89%)	11 (4.31%)	
Right	12251 (92.14%)	232 (90.98%)	
Not a paired site	506 (3.81%)	10 (3.92%)	
Histology recode			0.71
Follicular carcinoma	566 (4.26%)	13 (5.1%)	
Oxyphilic adenocarcinoma	572 (4.3%)	12 (4.71%)	
Papillary adenocarcinoma	11895 (89.46%)	223 (87.45%)	
others	263 (1.98%)	7 (2.75%)	
T stage			<0.001
T0	22 (0.17%)	1 (0.39%)	
T1	7931 (59.65%)	136 (53.33%)	
T2	2795 (21.02%)	45 (17.65%)	
T3	1940 (14.59%)	34 (13.33%)	
T4	468 (3.52%)	36 (14.12%)	
TX	140 (1.05%)	3 (1.18%)	
N stage			0.42
N0	8726 (65.63%)	168 (65.88%)	
N1	3863 (29.05%)	78 (30.59%)	
NX	707 (5.32%)	9 (3.53%)	
Radiation recode			0.34
None/Unknown	8954 (67.34%)	164 (64.31%)	
Yes	4342 (32.66%)	91 (35.69%)	
Chemotherapy recode			0.589
No/Unknown	13112 (98.62%)	253 (99.22%)	
Yes	184 (1.38%)	2 (0.78%)	
Surgery			0.802
None	483 (3.63%)	8 (3.14%)	
Yes	12813 (96.37%)	247 (96.86%)	
Tumor Size			0.033
<2	7789 (58.58%)	132 (51.76%)	
≥2cm	5507 (41.42%)	123 (48.24%)	
Bone and/or lung metastasis			<0.001
No	13033 (98.02%)	223 (87.45%)	
Yes	263 (1.98%)	32 (12.55%)	

**Table 2:** Baseline characteristics of thyroid cancer patients with and without bone and/or lung metastasis in SEER database.

Categories	Without bone and/or lung metastasis (N = 13033)	With bone and/or lung metastasis (N = 263)	*P-*value
Age (years)	50.36 ± 16.18	64.39 ± 17.16	<0.001
Race			<0.001
Black	761 (5.84%)	25 (9.51%)	
Other	2129 (16.34%)	65 (24.71%)	
White	10143 (77.82%)	173 (65.78%)	
Sex			<0.001
Female	9555 (73.31%)	142 (53.99%)	
Male	3478 (26.69%)	121 (46.01%)	
Marital status			<0.001
Divorced	909 (6.97%)	21 (7.98%)	
Separated	98 (0.75%)	3 (1.14%)	
Unmarried	157 (1.2%)	2 (0.76%)	
Widowed	524 (4.02%)	38 (14.45%)	
Married	8212 (63.01%)	141 (53.61%)	
Single	3133 (24.04%)	58 (22.05%)	
Household income			<0.001
<$60000	650 (4.99%)	8 (3.04%)	
$60000 - $79,999	2731 (20.95%)	49 (18.63%)	
$80000 - $119,999	7965 (61.11%)	145 (55.13%)	
$120,000+	1687 (12.94%)	61 (23.19%)	
Year of diagnosis			0.684
2018	3366 (25.83%)	62 (23.57%)	
2019	3406 (26.13%)	70 (26.62%)	
2020	2913 (22.35%)	66 (25.1%)	
2021	3348 (25.69%)	65 (24.71%)	
Laterality			0.325
Bilateral	154 (1.18%)	1 (0.38%)	
Left	379 (2.91%)	5 (1.9%)	
Right	500 (3.84%)	6 (2.28%)	
Not a paired site	12000 (92.07%)	251 (95.44%)	
Histology recode			<0.001
Follicular carcinoma	534 (4.1%)	32 (12.17%)	
Oxyphilic adenocarcinoma	259 (1.99%)	4 (1.52%)	
Papillary adenocarcinoma	11782 (90.4%)	113 (42.97%)	
others	458 (3.51%)	114 (43.35%)	
T stage			<0.001
T0	16 (0.13%)	6 (2.28%)	
T1	7905 (60.65%)	26 (9.89%)	
T2	2770 (21.25%)	25 (9.51%)	
T3	1854 (14.23%)	86 (32.7%)	
T4	368 (2.82%)	100 (38.02%)	
TX	120 (0.92%)	20 (7.6%)	
N stage			<0.001
N0	8641 (66.3%)	85 (32.32%)	
N1	3704 (28.42%)	159 (60.46%)	
NX	688 (5.28%)	19 (7.22%)	
Radiation recode			<0.001
None/Unknown	8851 (67.91%)	103 (39.16%)	
Yes	4182 (32.09%)	160 (60.84%)	
Chemotherapy recode			<0.001
No/Unknown	12923 (99.16%)	189 (71.86%)	
Yes	110 (0.84%)	74 (28.14%)	
Surgery			<0.001
None	368 (2.82%)	115 (43.73%)	
Yes	12665 (97.18%)	148 (56.27%)	
Tumor Size			<0.001
<2cm	7746 (59.43%)	43 (16.35%)	
≥2cm	5287 (40.57%)	220 (83.65%)	

**Figure 2:**
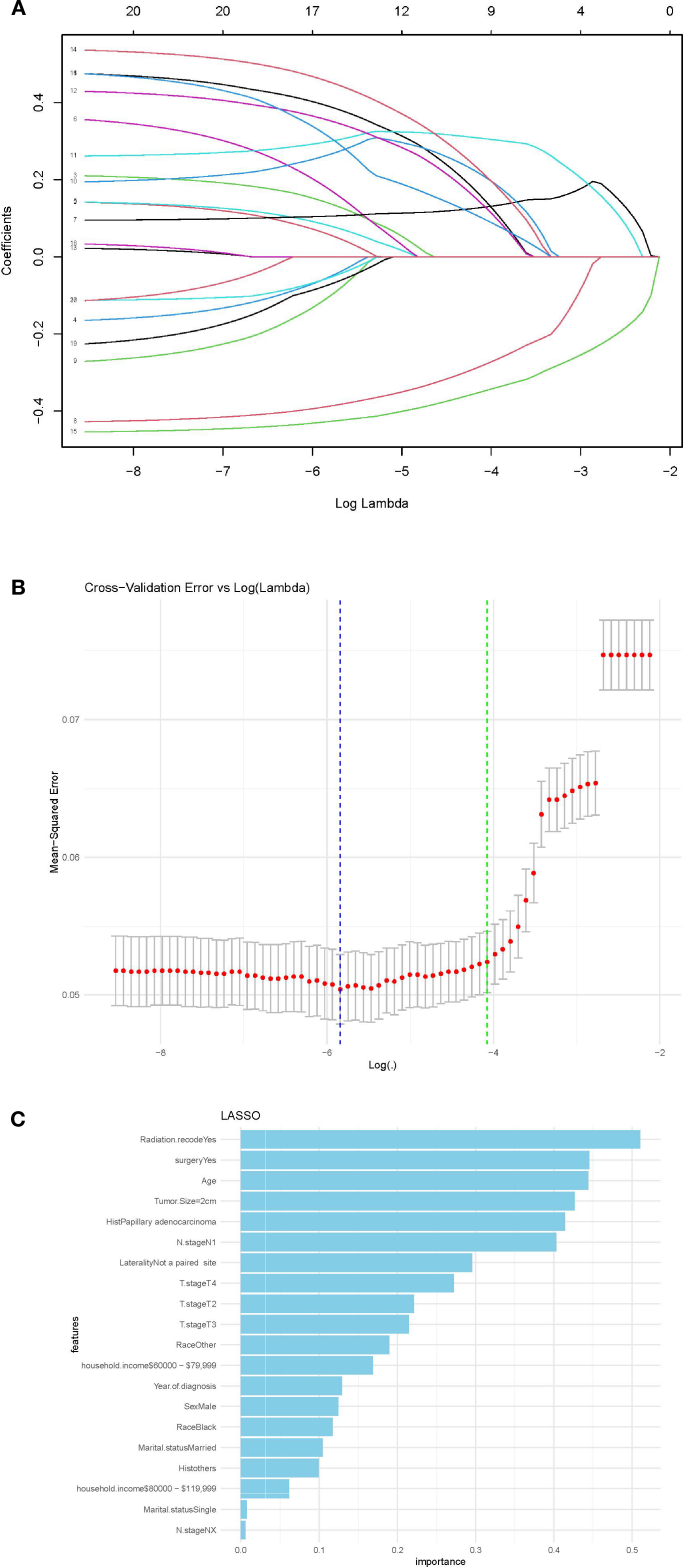
**(A)** LASSO regression coefficients shrinkage path; **(B)** Stability of features in LASSO regression,**(C)** Feature importance in predictive model in LASSO model.

The analysis ultimately narrowed down to nine key variables for inclusion in the final predictive model. These variables were selected based on their stability across the regularization path and their significant contribution to minimizing the cross-validation error, reflecting their strong predictive power regarding metastasis occurrence in DTC patients (**Figures 2A, B**). These selected features likely include some of the most prominent factors shown in the feature importance plot (**Figure 2C**), such as radiation, surgery, age, and tumor size, which are known to be critical in the prognosis and progression of DTC.

**Figure 3:**
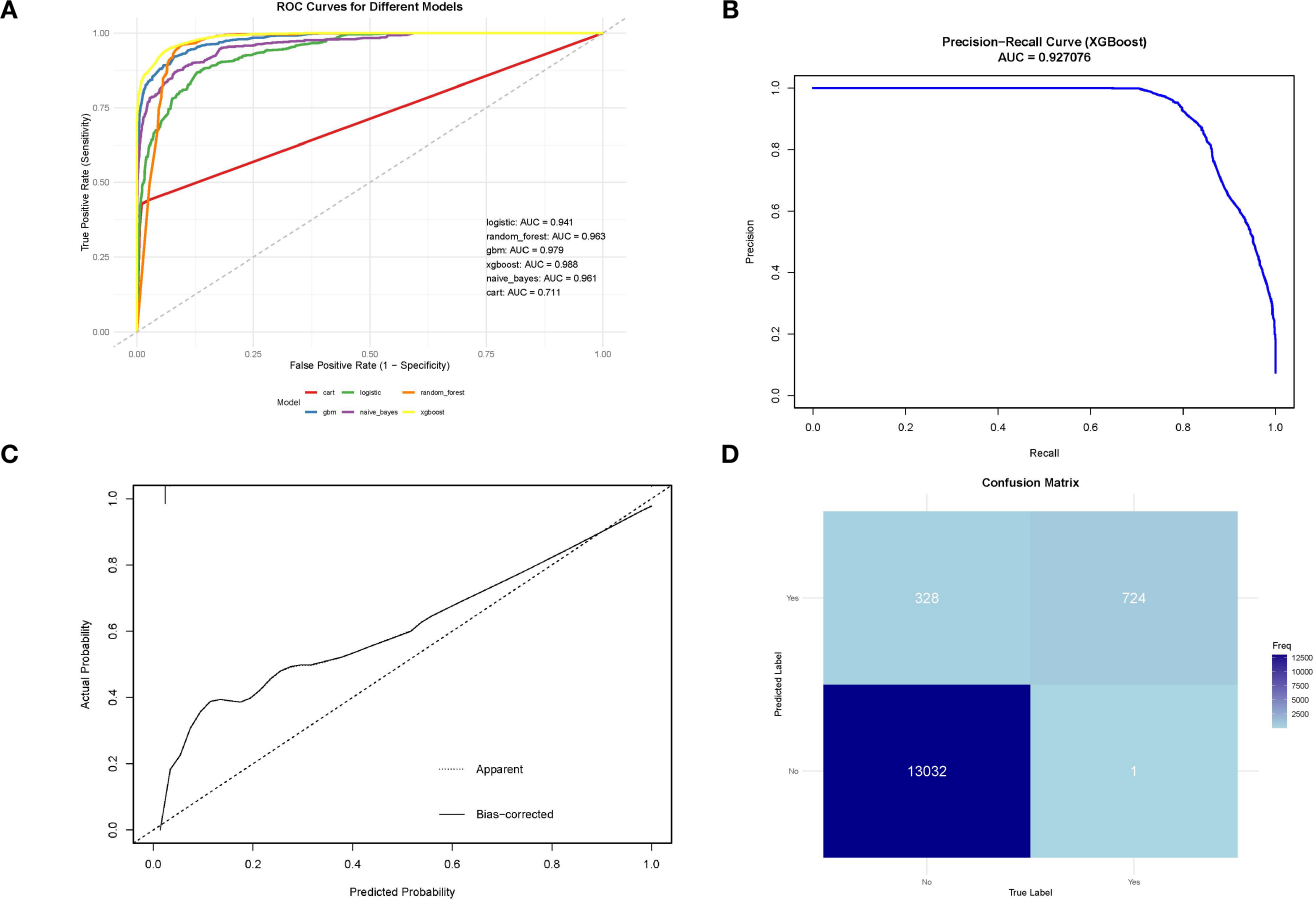
**(A)** ROC curve for machine learning model on SEER training data;**(B)**Precision-recall curve for XGBoost model on SEER training data;**(C)** Calibration curve of the XGBoost model;confusion matrix for XGBoost model on SEER training data.

**Figure 4:**
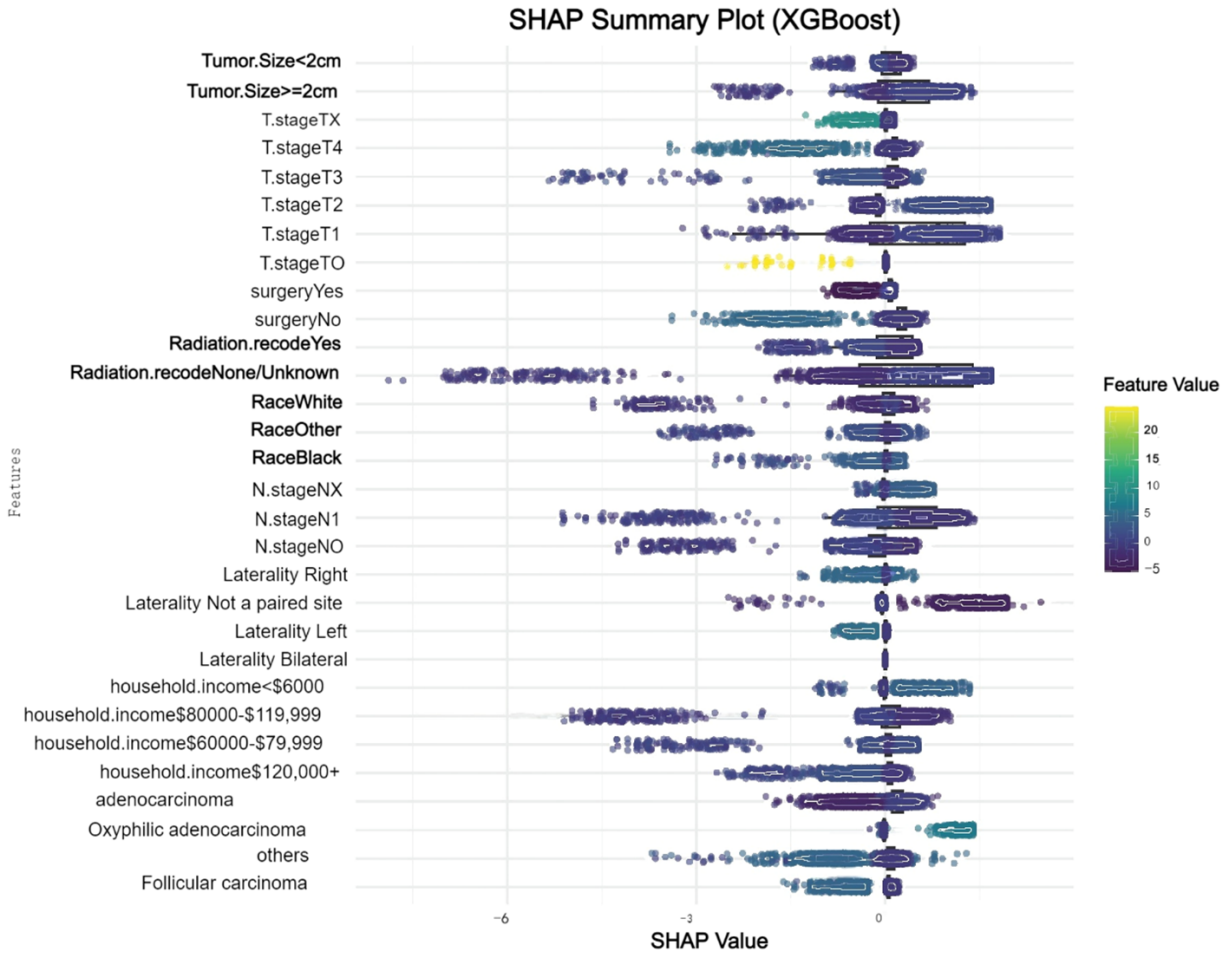
SHAP summary plot for feature influence in XGBoost model.

We conducted a comprehensive analysis of seven machine learning algorithms, comparing their performance based on accuracy, precision, recall, F1 score, and AUC. In line with previous research, models trained using oversampling techniques consistently outperformed those trained with undersampling. The detailed performance metrics for each machine learning model are presented in **Table 3**. Across all oversampled models, the AUC exceeded 0.800, with XGBoost achieving the highest performance, showing an AUC of 0.988 (95% CI: 0.986-0.991) on the training set (**Figure 3A**). A comparison of AUC values between XGBoost and traditional logistic regression demonstrated that XGBoost provided significantly higher diagnostic accuracy and predictive power. Moreover, the precision-recall curve for the XGBoost model exhibited an AUC of 0.927, underscoring its superior performance in managing the imbalanced dataset, where metastatic cases are underrepresented (**Figure 3B**). **Figure 3C** illustrates the calibration curve of the XGBoost model, indicating excellent agreement between predicted probabilities and observed outcomes, suggesting robust calibration. **Figure 3D** presents the confusion matrix for the XGBoost model. The model accurately identified 328 metastatic cases (true positives) and 13,032 non-metastatic cases (true negatives), though it misclassified only 1 metastatic patient as non-metastatic (false negatives). This confusion matrix underscores the model’s strong overall classification accuracy in distinguishing between metastatic and non-metastatic patients. The SHAP summary plot displayed here provides insights into the contribution of different features to the predictive model powered by XGBoost for bone and/or lung metastasis in DTC patients (**Figure 4**). Tumors size≥2cm is the most impactful feature, where larger values (indicated by the rightward extension of the blue dots) significantly increase the model’s prediction towards a higher likelihood of metastasis. Smaller tumor sizes (Tumor size<2cm) have less impact and are mostly associated with a lower risk prediction.

**Table 3:** Performance metrics of machine learning models for predicting bone and/or lung metastasis in DTC patients.

Model	Accuracy	Sensitivity	Specificity	F1_Score	AUC
Training set					
Logistic	0.95	0.99	0.451	0.974	0.941
Random forest	0.83	0.817	0.991	0.899	0.963
Gbm	0.973	0.995	0.702	0.986	0.979
Xgboost	0.977	1	0.688	0.988	0.988
Naive bayes	0.95	1	0.335	0.974	0.961
Cart	0.948	0.99	0.429	0.972	0.711
Validation set					
Xgboost	0.779	0.796	0.65	0. 91	0.866

**Figure 5:**
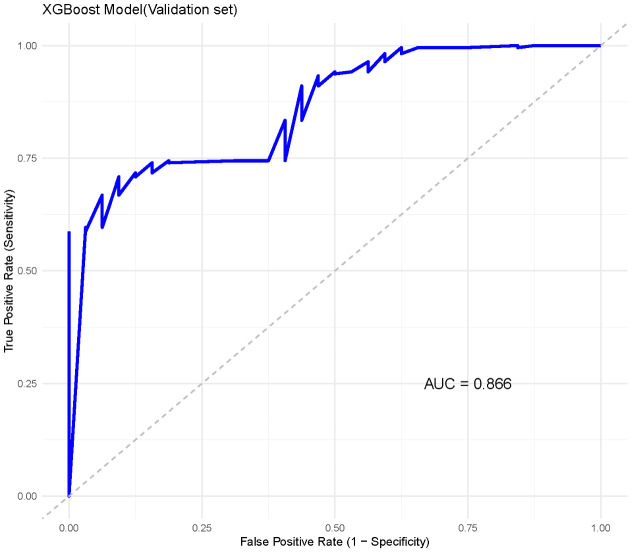
Precision-recall curve for XGBoost model in external validation.

In the external validation set, XGBoost demonstrated similarly strong performance, achieving an AUC of 0.866 (95% CI: 0.863–0.869) (**Figure 5**). The other indicators indicate that the XGBoost model shows a balanced performance, with relatively high sensitivity, making it effective in identifying positive cases of metastasis. However, specificity is moderate, suggesting that some negative cases may be incorrectly classified as positive. Overall, the model performs well, with a high F1 score (0. 91)and AUC, highlighting its effectiveness on the validation data.

**Figure 6:**
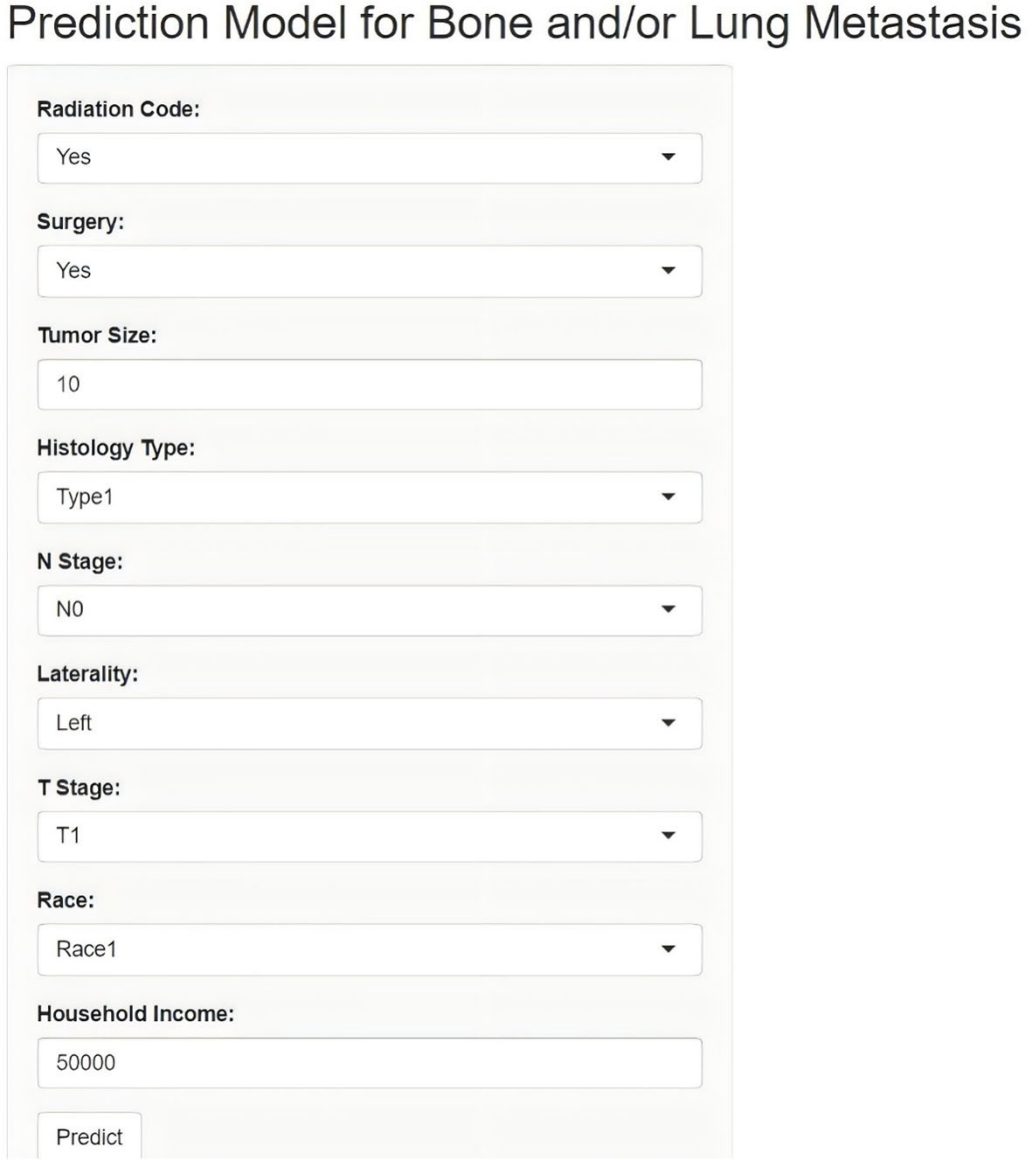
Online calculator for bone and/or lung metastasis prediction.

Lastly, this study developed an online network calculator for evaluating the risk of bone and/or lung metastasis in DTC patients, which can be applied to clinical patients (**Figure 6**). (http://127.0.0.1:3384).

## 4 Discussion

This study presents a machine learning-based model developed to predict bone and/or lung metastasis in DTC patients, leveraging data from the SEER database and validated with an independent cohort from China. Among the various algorithms explored, the XGBoost model demonstrated the most robust predictive power, particularly after using the SMOT to address the class imbalance inherent in metastasis data. This adjustment enhanced the model’s sensitivity and overall accuracy, positioning it as a powerful tool for identifying metastatic risk. Additionally, SHAP analysis identified tumor size, radiation therapy, and surgical interventions as primary factors influencing metastatic risk, highlighting the importance of these clinical variables in model interpretability.

In-depth analysis of the model’s key variables underscores its predictive capability (9). Tumor size emerged as the most influential factor, with larger tumors strongly linked to an increased risk of metastasis. This finding aligns with clinical evidence that associates greater tumor burden with more aggressive disease and poorer outcomes. This correlation between tumor size and metastatic risk could be due to the biological behavior of larger tumors, which may exhibit greater vascular and lymphatic involvement, thereby facilitating the spread of cancer cells. Radiation therapy and surgical interventions also proved to be significant predictors, likely due to the complex interplay between treatment modalities and disease progression (10–12). Notably, patients who underwent specific surgical procedures or received radiation showed different metastatic risk profiles, suggesting that tailored treatment approaches based on individual patient characteristics may be essential in optimizing outcomes. Additionally, tumor staging variables, such as T and N stages, were identified as critical factors, reflecting their well-established role in cancer staging and prognosis. Insights from SHAP values not only improve model transparency but also enhance the alignment of our findings with known clinical determinants of metastasis, thus reinforcing the reliability and relevance of our approach in a clinical setting.

Our study builds on and extends previous research in several key aspects. For instance, Mourad et al. applied machine learning to SEER data using feature selection algorithms to predict DTC prognosis, achieving an accuracy of 94.5% with a multilayer perceptron model (13). While Mourad et al. focused on overall survival, our study directly targets metastatic risk and applies a broader set of machine learning models, leveraging ensemble methods like XGBoost, which demonstrated enhanced predictive performance for metastasis. Furthermore, our use of SHAP values substantially improves model interpretability, offering a more nuanced understanding of feature importance—an aspect less emphasized in Mourad et al.’s work. In another study, Liu et al. developed models using SEER data to predict lung metastasis in DTC, with the RF model performing best, achieving an accuracy and area under the curve (AUC) of 0.99 (14). However, their study focused solely on lung metastasis, whereas our model provides a comprehensive assessment by predicting both bone and lung metastasis risk. Moreover, we conducted an external validation with a clinical cohort from China, which adds robustness and supports the generalizability of our findings—a validation step absent in Liu et al.’s study. Qiao et al. also explored multiple machine learning algorithms, including RF and XGBoost, to predict distant metastasis in DTC, with RF demonstrating strong performance (AUC of 0.960) (15). Our study aligns with these findings but goes further by employing SMOTE to address class imbalance and utilizing SHAP values for feature importance analysis, providing a more in-depth understanding of model behavior. Compared to these studies, our approach includes a broader range of clinical variables and employs LASSO-based feature selection, enhancing the model’s ability to capture the multifactorial nature of metastatic development in DTC. Furthermore, the use of an external validation cohort from a different population underscores the generalizability of our model, which is essential for clinical applicability. Unlike previous studies that often rely on a single dataset, our approach ensures broader applicability and reliability across diverse clinical settings, an advancement critical for real-world implementation.

Despite the strengths of our approach, several limitations must be acknowledged. First, as a retrospective study relying on SEER data, there is an inherent risk of bias associated with data collection and reporting. For instance, missing or incomplete records necessitated the exclusion of some patients, which may impact the overall representativeness of the sample and limit the generalizability of our findings to other populations. Second, the SEER database lacks detailed histopathological and molecular information, making it impossible to accurately identify and separately analyze subtypes such as high-grade differentiated thyroid carcinoma (high-grade DTC) and poorly differentiated thyroid carcinoma (PDTC). Given the biological and prognostic differences between these subtypes and conventional DTC, the inability to distinguish them represents a meaningful limitation. Additionally, important pathological variables such as extra-nodal extension (ENE)—a recognized risk factor for disease recurrence—are not consistently recorded in the SEER dataset and were therefore not included in our analysis. The absence of such features may limit the model’s ability to fully capture tumor aggressiveness and recurrence potential. Future studies incorporating institutional or prospective databases that provide access to detailed histological grading, mitotic index, tumor necrosis, ENE status, and molecular markers are warranted to refine model precision and enhance clinical applicability. Additionally, while SMOTE was employed to balance class distribution, synthetic data generation carries the risk of introducing noise or even overfitting, particularly in highly heterogeneous patient groups where subtle variations could affect model stability. Our use of an external validation cohort from the First Affiliated Hospital of Nanchang University and the First Hospital of Putian certainly adds robustness to our findings, yet the relatively small size of this cohort limits our ability to fully evaluate model performance across a broader range of clinical presentations. Finally, our model relies exclusively on clinical and demographic variables without incorporating molecular or genetic markers, which may limit its capacity to account for the biological heterogeneity of DTC metastasis. Looking forward, future studies could benefit from including larger and more diverse validation cohorts, ideally incorporating multiple international datasets to confirm the model’s robustness across a wider range of clinical and demographic profiles. Expanding these cohorts would not only enhance the statistical power of the analysis but also improve the model’s generalizability, which is crucial for its potential integration into clinical practice. Additionally, incorporating molecular biomarkers—such as genetic mutations, protein expression profiles, and epigenetic changes—could significantly enhance the predictive accuracy of machine learning models for metastatic risk in DTC. Multi-omics approaches may help overcome some current limitations by offering a more comprehensive view of disease biology and enabling the model to capture subtle biological patterns that purely clinical or demographic data may miss.

In practical terms, the development of a user-friendly online tool based on our model could facilitate the integration of machine learning into clinical workflows. Such a tool would allow clinicians to assess metastatic risk quickly, enabling more personalized treatment planning and potentially improving patient outcomes. However, while our model shows promise, further prospective validation in real-world clinical settings will be necessary to confirm its clinical utility and effectiveness. Prospective studies could evaluate how incorporating this tool into clinical decision-making processes impacts treatment strategies, patient management, and outcomes. Additionally, prospective testing may reveal new insights into model performance under diverse, dynamic clinical conditions, contributing to iterative improvements and refinements.

## 5 Conclusion

In conclusion, our study represents a significant step forward in leveraging machine learning to predict metastatic risk in DTC patients. By integrating SHAP values for feature interpretability and validating the model with an independent cohort, we have developed a robust and transparent predictive tool with potential clinical relevance. Addressing current limitations, such as expanding external validation and incorporating molecular data, could further enhance the model’s utility. As we continue to advance in the era of precision medicine, models like ours lay the groundwork for the next generation of predictive tools, supporting clinicians in providing more targeted, personalized care for DTC patients facing the risk of metastasis.

## Data Availability

The original contributions presented in the study are included in the article/supplementary material. Further inquiries can be directed to the corresponding author.

## References

[1] Chen DW Lang BHH McLeod DSA Newbold K Haymart MR. Thyroid cancer. Lancet. (2023) 401:1531–44. doi: 10.1016/S0140-6736(23)00020-X, PMID: 37023783

[2] Boucai L Zafereo M Cabanillas ME. Thyroid cancer: A review. Jama. (2024) 331:425–35. doi: 10.1001/jama.2023.26348, PMID: 38319329

[3] Rajan N Khanal T Ringel MD. Progression and dormancy in metastatic thyroid cancer: concepts and clinical implications. Endocrine. (2020) 70:24–35. doi: 10.1007/s12020-020-02453-8, PMID: 32779092 PMC7530083

[4] Wang LY Ganly I. Post-treatment surveillance of thyroid cancer. Eur J Surg Oncol. (2018) 44:357–66. doi: 10.1016/j.ejso.2017.07.004, PMID: 28754228 PMC7422645

[5] Janjua N Wreesmann VB. Aggressive differentiated thyroid cancer. Eur J Surg Oncol. (2018) 44:367–77. doi: 10.1016/j.ejso.2017.09.019, PMID: 29169931

[6] Greener JG Kandathil SM Moffat L Jones DT. A guide to machine learning for biologists. Nat Rev Mol Cell Biol. (2022) 23:40–55. doi: 10.1038/s41580-021-00407-0, PMID: 34518686

[7] Deo RC. Machine learning in medicine. Circulation. (2015) 132:1920–30. doi: 10.1161/CIRCULATIONAHA.115.001593, PMID: 26572668 PMC5831252

[8] Handelman GS Kok HK Chandra RV Razavi AH Lee MJ Asadi H. eDoctor: machine learning and the future of medicine. J Intern Med. (2018) 284:603–19. doi: 10.1111/joim.12822, PMID: 30102808

[9] Reiners C Drozd VM. Editorial: differentiated thyroid cancer - risk adapted therapy, genetic profiling and clinical staging. Front Endocrinol (Lausanne). (2021) 12:755323. doi: 10.3389/fendo.2021.755323, PMID: 34690935 PMC8531744

[10] Agosto Salgado S Kaye ER Sargi Z Chung CH Papaleontiou M. Management of advanced thyroid cancer: overview, advances, and opportunities. Am Soc Clin Oncol Educ Book. (2023) 43:e389708. doi: 10.1200/EDBK_389708, PMID: 37186883

[11] van Velsen EFS Leung AM Korevaar TIM. Diagnostic and treatment considerations for thyroid cancer in women of reproductive age and the perinatal period. Endocrinol Metab Clin North Am. (2022) 51:403–16. doi: 10.1016/j.ecl.2021.11.021, PMID: 35662449

[12] Robbins J Merino MJ Boice JD Jr. Ron E Ain KB Alexander HR. Thyroid cancer: a lethal endocrine neoplasm. Ann Intern Med. (1991) 115:133–47. doi: 10.7326/0003-4819-115-2-133, PMID: 2058861

[13] Mourad M Moubayed S Dezube A Mourad Y Park K Torreblanca-Zanca A. Machine learning and feature selection applied to SEER data to reliably assess thyroid cancer prognosis. Sci Rep. (2020) 10:5176. doi: 10.1038/s41598-020-62023-w, PMID: 32198433 PMC7083829

[14] Liu W Wang S Ye Z Xu P Xia X Guo M. Prediction of lung metastases in thyroid cancer using machine learning based on SEER database. Cancer Med. (2022) 11:2503–15. doi: 10.1002/cam4.4617, PMID: 35191613 PMC9189456

[15] Qiao L Li H Wang Z Sun H Feng G Yin D. Machine learning based on SEER database to predict distant metastasis of thyroid cancer. Endocrine. (2024) 84:1040–50. doi: 10.1007/s12020-023-03657-4, PMID: 38155324

